# Transcriptomics and Metabolomics Reveal Biosynthetic Pathways and Regulatory Mechanisms of Phenylpropanes in Different Ploidy of *Capsicum frutescens*

**DOI:** 10.3390/plants13233393

**Published:** 2024-12-03

**Authors:** Yinxin Yang, Qihang Cai, Yanbo Yang, Xuan Wang, Liping Li, Zhenghai Sun, Weiwei Li

**Affiliations:** 1 Yunnan International Joint R&D Center for Intergrated Utilization of Ornamental Grass, College of Landscape and Horticulture, Southwest Forestry University, Kunming 650224, China; yangyinxin@swfu.edu.cn (Y.Y.); caiqihang_98@163.com (Q.C.); yangyanbo_99@163.com (Y.Y.); wangxuan@swfu.edu.cn (X.W.); 2 College of Geography and Ecotourism, Southwest Forestry University, Kunming 650224, China; 3 Yunnan International Joint Center of Urban Biodiversity, Kunming 650223, China

**Keywords:** *Capsicum frutescens*, polyploid, transcriptome, metabolome, phenylalanine

## Abstract

Pepper is a significant cash crop, and *Capsicum frutescens* is an exemplary variety of pepper cultivated for its distinctive flavor and substantial nutritional value. Polyploidization of plants often leads to an increase in their biomass and improved stress tolerance, and thus has important applications in plant breeding and improvement. In this study, germplasm innovation was carried out by polyploidy induction of *C. frutescens* by colchicine. To investigate the effects of polyploidization on *C. frutescens*, we conducted transcriptomic and metabolomic analyses of diploids and homotetraploids of *C. frutescens* to gain insights into the mechanisms of metabolite composition and molecular regulation of *C. frutescens* by polyploidization. Based on the analysis of metabolomics and transcriptomics data, a total of 551 differential metabolites were identified in the leaves of *C. frutescens* of different ploidy and 634 genes were significantly differentially expressed. In comparison, 241 differential metabolites and 454 genes were significantly differentially expressed in the mature fruits of *C. frutescens* of different ploidy. Analysis of KEGG enrichment of differentially expressed genes and differential metabolites revealed that both differential metabolites and differentially expressed genes were highly enriched in the phenylalanine metabolic pathway. It is worth noting that phenylpropanoids are highly correlated with capsaicin synthesis and also have an effect on fruit development. Therefore, we comprehensively analyzed the phenylalanine metabolic pathway and found that chromosome doubling significantly down-regulated the expression of genes upstream of phenylalanine (*PAL*, *4CL*), which promoted lignin accumulation, and we suggested that this might have led to the enlargement of polyploid *C. frutescens* fruits. This study provides some references for further research on the phenotypic traits of different ploidy of *C. frutescens*, cloning of key regulatory genes, and using genetic engineering techniques in *C. frutescens* breeding for germplasm improvement.

## 1. Introduction

Polyploid is a heritable individual containing three or more chromosome groups in somatic cells. It exists in both animals and plants and is more common in higher plants [1]. Polyploidized plants often exhibit new phenotypes, such as larger vegetative organs, increased metabolites, and other characteristics that were not present in their ancestral diploids. However, the adaptive advantage of these mutations to environmental change is important from the point of view of the evolution of species [2,3]. Genome sequencing data of *Oryza sativa*, *Arabidopsis thaliana*, and *Vitis vinifera*, which were once considered to be classical diploid plants, indicate that their genomes contain ancient polyploid markers [4]. It has been shown in the literature that ancient plant genome doubling preceded the differentiation of monocotyledons and true dicotyledons [5], which includes Solanaceae [6].

Plant polyploidy has a significant impact on the genome. Not only does it result in genome reorganization, but it also leads to changes in gene expression, and epigenetic and transcription factor activity [7]. There is a large number of studies showing that genetic changes and epigenetic differences often occur in the early stages of polyploid formation [8,9,10,11,12,13,14]. Nonadditive gene expression is a common feature in plant polyploids. Transcriptome sequencing analysis of different plant groups shows that changes in gene expression are common in newborn polyploid plants [15,16,17,18,19]. However, the extent of the change depends largely on the specific polyploid species. A large number of studies in recent years have shown that polyploidization has a relatively small effect on the relative expression levels of genes. In polyploid citrus *Citrus limon*, *Zea mays*, and *Arabidopsis thaliana*, there are only a few differential genes [20,21,22,23]. In particular, only 10% of the genes in the homologous polyploid study of *Solanum phureja* (1×, 2×, 4×) showed differential expression levels, and most of the differential genes were detected in haploids [24]. Pignatta et al. had assumed that polyploidy would change a large number of genomes, but it turned out that only a small fraction of the genes were changed [20]. This phenomenon was also confirmed in the studies of Yu et al. on *Arabidopsis thaliana*, where, to cope with the effects of polyploidization on the genome, there may be homopolyploids that respond in a way that is similar to that of heteropolyploids but with less impact on the genome [25]. Polyploidy may affect many plant traits such as plant morphology, enzyme activity, plant stress tolerance, and its metabolite content [23,26]. Mishra et al.’s research also illustrates this view, showing that polyploidization can make the Papaver somniferum produce more morphine [27]. Polyploidized *Populus ussuriensis* phenylalanine compounds are also metabolized more vigorously [28].

The phenylalanine metabolic pathway is one of the most important metabolic pathways in plants, producing more than 8000 metabolites that play important roles in plant growth and development and environmental adaptation [28]. Phenylpropanoids contain a phenyl group attached to the 3-c propane side chain, and a change in the position of the substituent on the benzene ring and the double bond with the propylene group can produce a large number of biologically active compounds [29,30]. Chemical modification of the basic backbone of phenylpropanoid is achieved by a series of enzymes, such as oxygenases, oxidoreductases, ligases, and various transferase enzymes, which makes phenylpropanoid biosynthesis diverse. Branches of phenylpropanoid metabolism can produce a variety of downstream products, such as flavonoids, hydroxycinnamates, and hydroxycinnamic acid amides (HCAAs). It is worth noting that lignin, which is necessary for the mechanical support of plant growth [31], is a polymer synthesized from phenylpropanoids, and polyploid plants usually have enlarged leaves, fruits, and other organs, which require more lignin support [32]. Phenylpropanoids not only have a large impact on plant development but also play an important role in plant stress tolerance [33,34]. In studies on maize, it has been reported that up-regulation of the *ZmCCoAOMT2* gene leads to enhanced lignification, which improves disease resistance [35]. In addition, silencing of the *GhCOMT* gene has been reported in cotton studies to reduce lignin synthesis and thus disease resistance in cotton [36]. Phenylpropanoids also play an important role in abiotic stresses, and recent studies have shown the up-regulation of gene expression upstream of the phenylalanine pathway such as *PAL* in tobacco [37]. Up-regulation of *CmCAD2* and *CmCAD3* in melon (*Cucumis melo*) leads to the accumulation of lignin, which contributes to plant response to drought stress [38]. Accumulation of phenylpropanoids due to *PAL* and *CAD* expression in manzanita and loquat (*Eribotrya japonica*) contributes to plant acclimatization to cold environments [39,40]. Therefore, analyzing differences in the phenylalanine substitution pathway is essential for analyzing the mechanism of formation of superior traits produced by plants after polyploidization [41,42].

Pepper is an important vegetable and spice crop that is widely cultivated worldwide. Peppers originated in Mexico, and the use of wild peppers dates back 6500 years [43]. Domestication of pepper is to remove the wild species from its origin and cultivate it artificially so that the wild species, which falls easily and has small fruits, a single color, and upward-facing fruits, can be bred into the cultivated species, which does not fall easily and has downward-facing fruits, a variety of shapes, bright colors, and good economic benefit [44]. Polyploidization can increase plant biomass and enlarge the fruits to obtain higher economic value, and the use of chemical reagents to make *C. frutescens* polyploidization can make *C. frutescens* fruits enlarged, solving the problem of the small biomass and the low economic value of *C. frutescens.* With the development of high-resolution mass spectrometry (LC-MS) and high-throughput sequencing technologies, it is possible to study the differences in metabolite profiles and molecular mechanisms of metabolic regulation in organisms, and the joint analysis of multi-omics technologies can identify the potential interactions between genes and metabolites, and explore the potential relationship between molecular mechanisms and metabolic pathways. In recent years, multi-omics techniques have been widely applied to phenotypic analysis of many plant species, such as camellia (*Camellia japonica*), cassava (*Manihot esculenta*), and other plants. Although phenylpropanoid metabolic pathways have been reported, joint metabolomic analysis of phenylpropanoid-related transcriptomes based on different ploidy pepper materials has rarely been reported. Therefore, it is important to study the joint analysis of transcriptomics and metabolomics data after the autopolyploidization of *C. frutescens*.

In this study, we conducted a comparative analysis of transcriptomics and metabolomics data of *C. frutescens* leaves and ripe *C. frutescens* fruits to understand the changes in phenylpropanoid metabolism in *C. frutescens* leaves and ripe *C. frutescens* fruits after polyploidization, and to identify DEGs associated with phenylpropanoid biosynthesis in *C. frutescens* of different ploidy. The results of transcriptomics and metabolomics analyses revealed the molecular regulatory mechanisms related to the accumulation of phenylpropanes in *C. frutescens* of different ploidy, laying a certain foundation for the subsequent breeding of *C. frutescens*.

## 2. Results

### 2.1. Identification of Different Ploidy of C. frutescens and Morphological Observations

**Figure 1:**
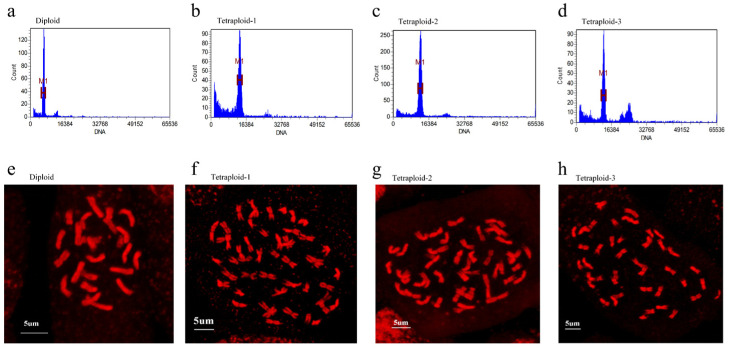
Identification of different ploidy of *C. frutescens*: (**a**) flow cytometry analysis of the third pair of true leaves of diploid *C. frutescens*; (**b**–**d**) flow cytometry analysis of the third pair of true leaves of tetraploid *C. frutescens* (3 biological replicates); (**e**) analysis of the apical root pressure of diploid *C. frutescens* seeds; (**f**–**h**) analysis of the apical root pressure of tetraploid *C. frutescens* seeds.

Common *C. frutescens* is diploid, with a chromosome number of 2n = 2x = 24. To identify the ploidy of the mutagenized plants, the young leaves were analyzed by flow cytometry. The results showed a significant increase in DNA content in the mutagenized plants compared to the control (Figure 1a–d). By observing the root tip pressings, it was found that all the cells in the control plants had 24 chromosomes and the mutagenized plants contained 48 chromosomes (Figure 1e–h).

**Figure 2:**
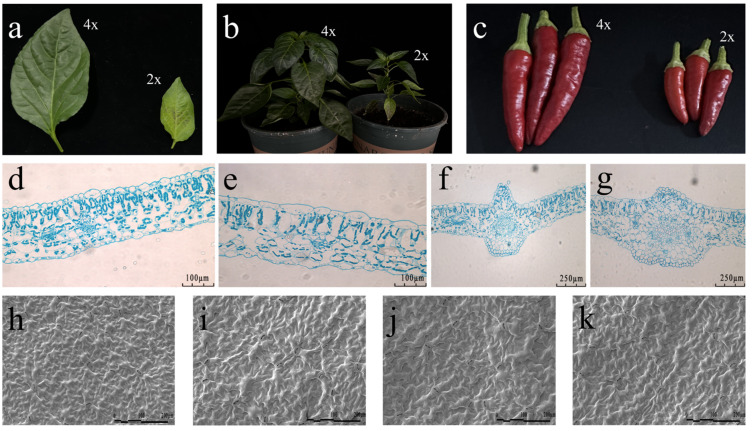
Morphological observations of different ploidy of *C. frutescens*: (**a**) comparison of the third pair of true leaves 50 d after planting of different ploidy of *C. frutescens* seeds; (**b**) comparison of the plants 70 d after planting of different ploidy of *C. frutescens* seeds; (**c**) comparison of the size of the fruits 80 d after flowering of different ploidy of *C. frutescens*; (**d**–**g**) analysis of the third pair of paraffin sections of true leaves 50 d after planting of different ploidy of *C. frutescens* seeds; (**h**–**k**) scanning electron microscopy analysis of the seeds of *C. frutescens* of different ploidy of *C. frutescens* and scanning electron microscope analysis of the third pair of true leaves 50 d after planting.

Compared with untreated *C. frutescens*, mutated *C. frutescens* leaves appeared significantly altered, with a deepening in leaf color and a thickening of leaves in the mutant strain (Figure 2a). Plants of the mutant strain were significantly larger than those of the control (Figure 2b), and there was significant enlargement of the fruits (Figure 2c). The palisade cells were significantly enlarged (Figure 2d,e), and the cross-sectional area of the leaf veins was significantly larger (Figure 2f,g). The number of stomata on the abaxial surface of the leaf was slightly reduced compared to the diploid, but the surface undulation was more pronounced (Figure 2h–k).

### 2.2. Metabolome Data Analysis of C. frutescens with Different Ploidy Levels

**Figure 3:**
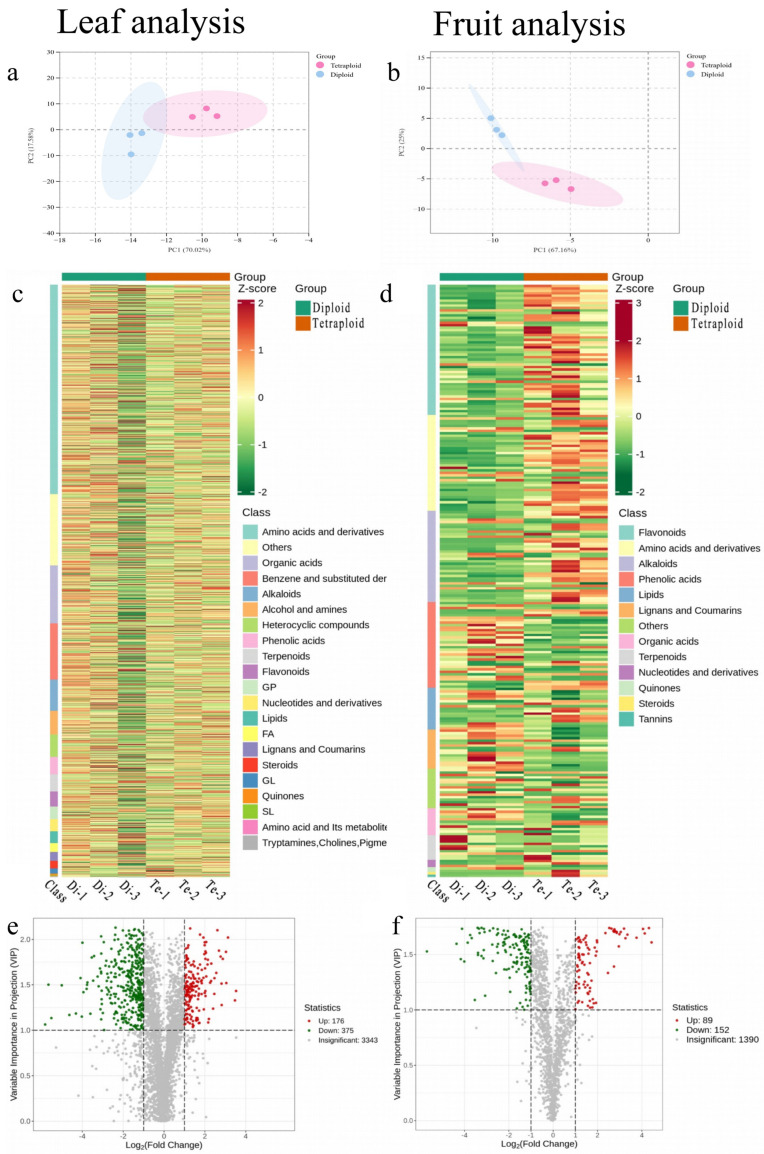

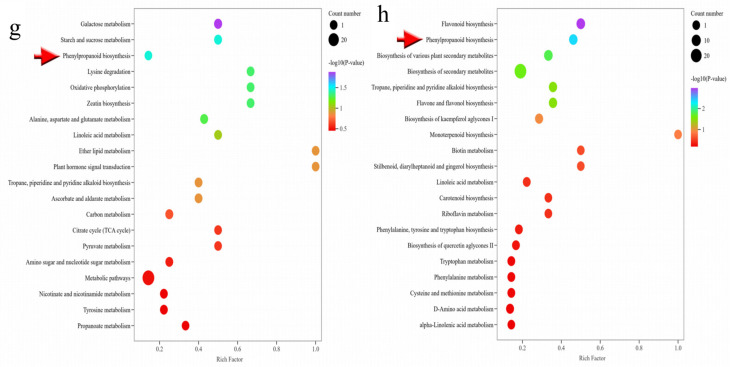
Metabolome analysis of different ploidy *C. frutescens* (“tetraploid” for diploid, “diploid” for tetraploid). The criteria for differential metabolite screening in the heat map were VIP (variable importance in the project) ≥ 1 and 0.5 ≤ fold change∣ ≥ 2. Red represents rising metabolite content, green represents falling metabolite content, and differential metabolite classification is shown on the right. Each point in the volcano diagram represents a metabolite, where green points represent down-regulated difference metabolites, red points represent up-regulated difference metabolites, and grey points represent metabolites with insignificant differences; the abscissa represents the logarithmic value of the multiple of the difference in the relative content of a metabolite between the two groups of samples (log_2_FC), and the greater the absolute value of the abscissa, the greater the difference in the relative content of the substance between the two groups of samples. The vertical coordinate indicates the VIP value, and the larger the value of the vertical coordinate, the more significant the difference is and the more reliable the differential metabolite obtained from the screening. In the differential metabolite KEGG enrichment bubble plot, the abscissa Rich factor is the ratio of the number of differential metabolites in the corresponding pathway to the total number of metabolites annotated to that pathway, and a larger value indicates a larger degree of enrichment. The vertical coordinate is the name of the pathway; the color of the point is the *p*-value size, with more purple indicating more significant enrichment. The size of the point represents the number of differential metabolites enriched, and the larger the number the greater the number of metabolites. (**a**) Leaf metabolite PCA principal component analysis; (**b**) fruit metabolite PCA principal component analysis; (**c**) leaf differential metabolite analysis heat map; (**d**) fruit differential metabolite analysis heat map; (**e**) leaf differential metabolite volcano map; (**f**) fruit differential metabolite volcano map; (**g**) leaf differential metabolite KEGG-enriched bubble map (The red arrow points to the pathway “phenylpropanoid biosynthesis”.); (**h**) fruit differential metabolite KEGG-enriched bubble plot (The red arrow points to the pathway “phenylpropanoid biosynthesis”.).

Metabolite data were analyzed to evaluate overall differences between groups using PCA, a tool that uses a small number of principal components to reveal internal structure between multiple metabolic groups. The results showed three biological replicates clustered together for both diploid and tetraploid samples, indicating that the results were highly reproducible between each group. The large distances between the diploid and tetraploid groups reflect differences in metabolite variation with ploidy (Figure 3a,b).

To gain a comprehensive understanding of the metabolite composition in *C. frutescens* leaves and fruits of different ploidy, and to study the differences in metabolites between diploids and tetraploids, *C. frutescens* leaves were screened for their metabolic profiles using an untargeted metabolomics (NTM) approach. The metabolic profiles of *C. frutescens* fruits were screened by the UPLC-MS/MS method. A total of six *C. frutescens* leaf samples were selected and divided into two groups for metabolic studies, and a total of 3343 metabolites were detected. Comparative analyses yielded 176 up-regulated significant metabolites and 375 down-regulated significant metabolites. A total of six samples of *C. frutescens* ripe fruits were selected and divided into two groups for metabolic study. The comparative analysis yielded 241 significantly different metabolites, of which 152 metabolites were down-regulated and 89 metabolites were up-regulated. Categorical analysis of all detected metabolites showed that amino acids and their derivatives accounted for the largest proportion of all detected metabolites in *C. frutescens* leaves with different ploidy, while flavonoid compounds were detected in the largest proportion in *C. frutescens* fruits with different ploidy (Figure 3c–f).

KEGG database enrichment analysis was performed based on differential metabolite results. The analysis showed significant differences in metabolites accumulated in the leaves and fruits of *C. frutescens* of different ploidy, with differential metabolites being significantly enriched in multiple metabolic pathways (Figure 3g,h), and it is noteworthy that differential metabolites in both fruits and leaves were enriched in the phenylalanine metabolic pathway (ko00940).

### 2.3. Transcriptome Data Analysis of C. frutescens

To investigate the molecular mechanisms of differential metabolites, we sequenced the transcriptome of *C. frutescens* leaves and mature fruits of different ploidy, and the assay was filtered to obtain 100.02 Gb of clean data. All Q30 base percentages were ≥95%, and the sequencing results were reliable. With Zhangshugang_genome.fa.gz (http://ted.bti.cornell.edu/ftp/pepper/genome/Zhangshugang/) (accessed on 3 March 2024), a reference genome comparison, the comparison efficiency was 94.85–96.91% (Table 1). It indicates that the quality of this transcriptome sequencing was good enough for subsequent analysis.

**Figure 4:**
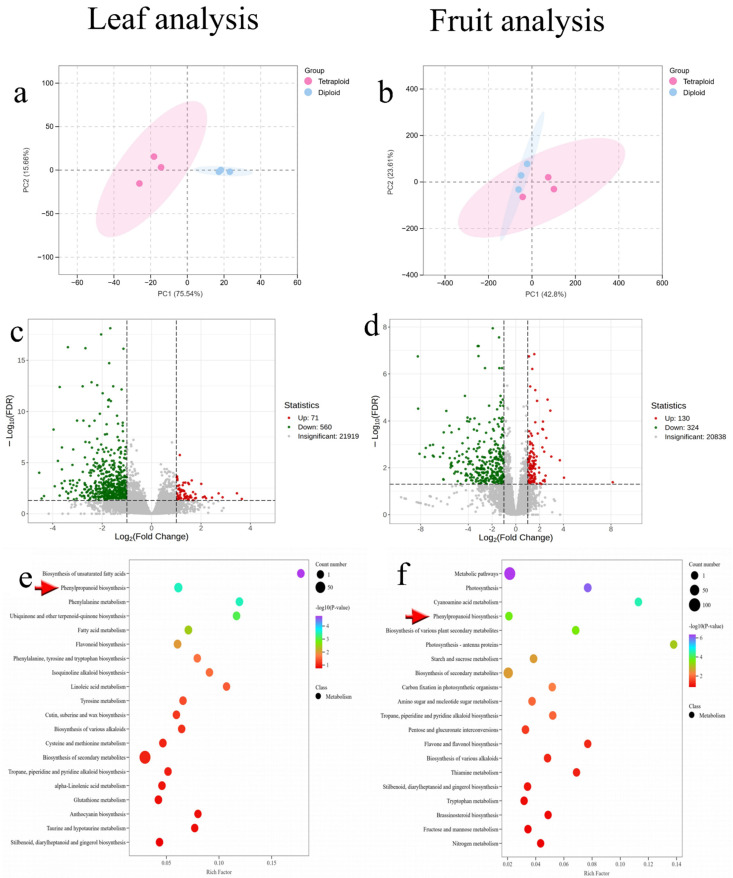
Transcriptome analysis of different ploidy of *C. frutescens* (“diploid” for diploid, “tetraploid” for tetraploid). Each dot in the volcano plot represents a gene, where green dots represent down-regulated genes, red dots represent up-regulated genes, and gray represents genes with insignificant differential expression; the abscissa indicates the gene expression fold change (log_2_FC), and the larger the absolute value of the abscissa, the larger the relative expression difference of the gene between the two groups of samples. The abscissa Rich factor in the differential gene KEGG enrichment bubble plot is the ratio of the number of differential genes in the corresponding pathway to the total number of genes annotated to the pathway, and a larger value indicates a greater degree of enrichment. The vertical coordinate is the name of the pathway; the color of the point is the *p*-value size, and the more purple it is, the more significant the enrichment is. The size of the point represents the number of genes enriched to the differential genes, and the bigger it is, the more genes there are. (**a**) PCA analysis of leaf differential genes; (**b**) PCA analysis of fruit differential genes; (**c**) volcanic map analysis of leaf differential genes; (**d**) volcano map analysis of fruit differential genes; (**e**) KEGG enrichment analysis of leaf differential genes (The red arrow points to the pathway “phenylpropanoid biosynthesis”.); (**f**) KEGG enrichment analysis of fruit differential genes (The red arrow points to the pathway “phenylpropanoid biosynthesis”.).

Differential gene expression sets were analyzed using PCA, and the results showed three biological replicates clustered together for both diploid and tetraploid samples, indicating that the results were highly reproducible between each group. The large distances between diploid and tetraploid populations reflect differences in gene expression levels with ploidy (Figure 4a,b).

A total of 631 differential genes were detected in different ploidy *C. frutescens* leaves, including 560 down-regulated genes and 71 up-regulated genes (Figure 4c). A total of 454 differential genes were detected in mature fruits of different ploidies of *C. frutescens*, including 324 down-regulated genes and 130 up-regulated genes (Figure 4d). Comparative analysis revealed that the number of down-regulated genes after polyploidization of *C. frutescens* was higher than the number of up-regulated genes.

KEGG enrichment analysis showed that the KEGG pathway was most significantly enriched in *C. frutescens* leaves of different ploidy for the biosynthesis of secondary metabolites, followed by the biosynthesis of phenylpropanoids and the biosynthesis of flavonoids (Figure 4e). The most significant enrichment of the KEGG pathway in mature fruits of different ploidy *C. frutescens* was the biosynthesis of secondary metabolites, followed by sucrose metabolism and the biosynthesis of phenylpropanoid compounds (Figure 4f). Both parts were enriched to varying degrees in the biosynthetic pathway of phenylalanine metabolism, which was selected for subsequent analysis in conjunction with previous studies.

### 2.4. Conjoint Transcriptome–Metabolome Analysis

Phenylpropanoids are important secondary metabolic pathways in plants and are highly correlated with the synthesis of lignin, lignans, and flavonoids. In tetraploid *C. frutescens* leaves, phenylalanine ammonia-lyase (*PAL*), coumarin acid ligase (*4CL*), and cinnamoyl reductase (*CCR*) genes were down-regulated to a certain extent compared to diploid *C. frutescens* leaves. Both caffeoyl-CoA O-methyltransferase (E2.1.1.104) and conifer aldehyde dehydrogenase (*REF1*) genes produced some degree of up-regulation of expression in tetraploid *C. frutescens* ripe fruits compared to diploid *C. frutescens* fruits. This change may have led to a rise in the accumulation of coniferyl alcohol in mature fruits of *C. frutescens*, thereby leading to differential expression of peroxidase (E1.11.1.7) in mature fruits of *C. frutescens*. These results suggest that *C. frutescens* chromosome doubling may affect developmental processes as well as phenylpropanoid biosynthesis by regulating phenylpropanoid biosynthesis-related gene expression.

**Figure 5:**
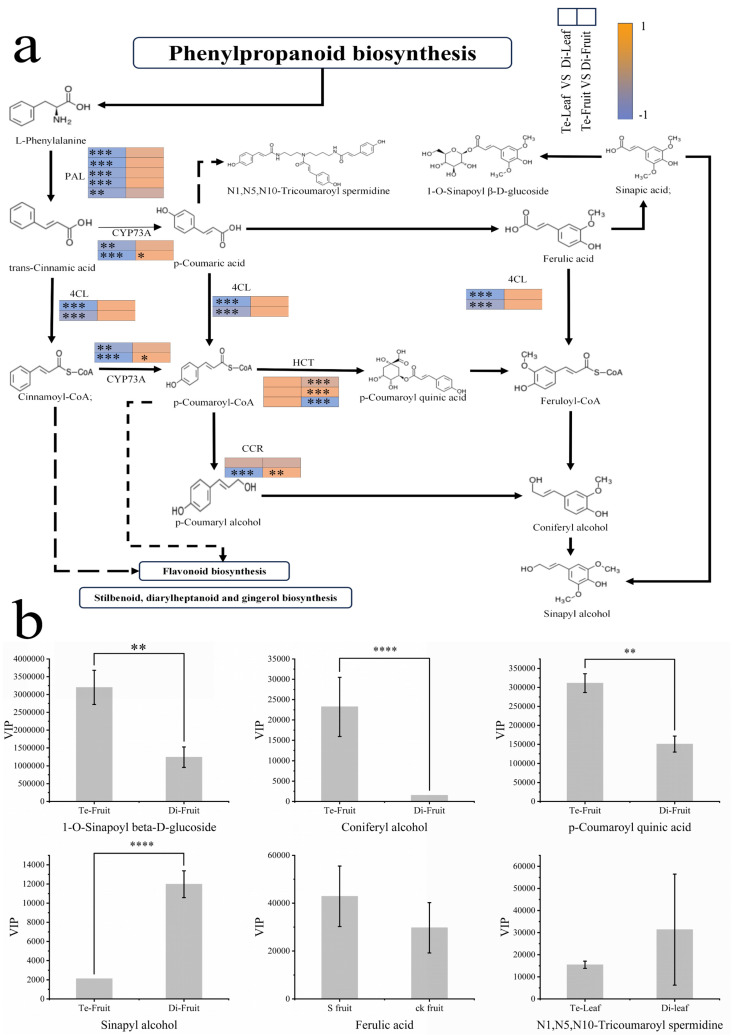

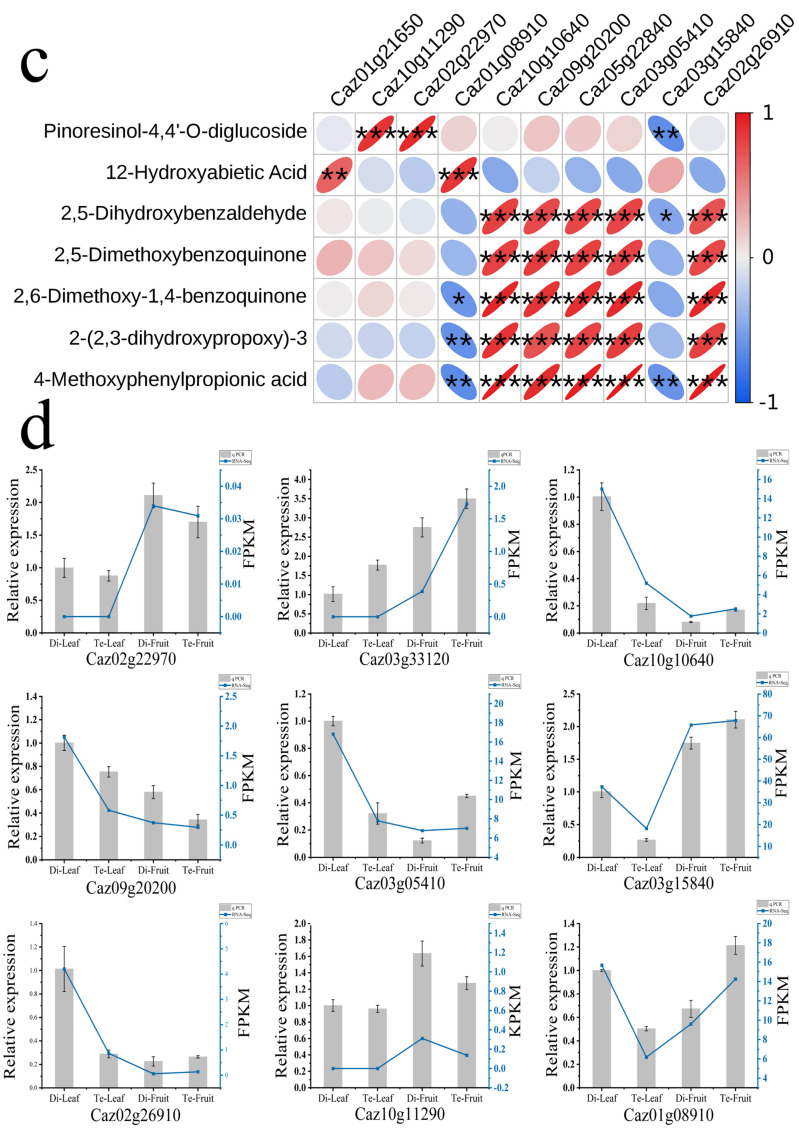
Conjoint metabolome–transcriptome analysis (“*” is significant (*p* < 0.05), “**” is very significant (*p* < 0.01), “***” is highly significant (*p* < 0.001), “****” is especially significant (*p* < 0.0001)). (**a**) Phenylalanine metabolic pathway analysis figure (heatmap in Log_2_FC, significance indicated by “*”); (**b**) differential phenylpropanoid compounds analysis figure (plotted with VIP values, three biological replicates were performed, significance is indicated by “*”. (“*” is significant (*p* < 0.05), “**” is very significant (*p* < 0.01), “***” is highly significant (*p* < 0.001)); (**c**) graph of differential phenylpropanoid compounds and phenylpropanoid metabolic pathway differential gene correlation analysis (abscissa is the gene ID, vertical axis is the metabolite name, and significance is indicated by “*”); (**d**) combined qRT-PCR and RNA-Seq analysis figure (relative gene expression was calculated using the 2^−ΔΔCT^ method, histograms indicate mean ± scale (three replicates), and folded lines indicate RNA-Seq. “Di-Leaf” indicates diploid leaves, “Te-Leaf” indicates tetraploid leaves, “Di-Fruit” indicates diploid fruits, and “Te-Fruit” indicates tetraploid fruit).

Based on the results of transcriptomics and widely targeted metabolomics analyses, differential metabolites as well as differential genes were mapped to the phenylpropanoid biosynthetic pathway to elucidate the relationship between phenylpropanoid synthesis and genes. More phenylpropanoid synthesis-related down-regulated genes than up-regulated genes were found in tetraploid *C. frutescens* compared to diploid *C. frutescens*. This result suggests that these down-regulated genes may have induced the production of more obtained phenylpropanoids and led to increased accumulation of phenylpropanoids. The integrated metabolite map shows that chromosome doubling may promote the accumulation of phenylpropanoid compounds (Figure 5a,b) by down-regulating the expression of genes upstream of the phenylpropanoid synthesis pathway (e.g., *PAL* and *4CL*), such as coumaroylquinic acid, ferulic acid, 1-O-alkynoyl-β-D-glucose, and coniferyl alcohol. We reasoned that these genes may negatively regulate the production of phenylpropanoid compounds.

Correlation analysis of differential genes on the phenylpropanoid compound pathway with differential metabolites showed that 10 differential genes were correlated with all 7 differential metabolites, with 29 positive and 6 negative correlations (Figure 5c). It is noteworthy that five of the differential genes (*PAL1*, *PAL2*, *PAL3*, *4CL1*, and E2.1.1.104) had high correlations with the differential metabolites, suggesting that these genes may be highly correlated with phenylpropanoid compounds synthesis. The phenylpropanoid compound differential genes were selected for qRT-PCR analysis to validate the RNA-Seq data, and the results showed that the qRT-PCR expression trend was consistent with the trend of the FPKM values from the transcriptomics analysis results (Figure 5d), further confirming the authenticity and reliability of the transcriptomics data.

## 3. Discussion

Polyploidization is considered an important driver of genome evolution and may provide new germplasm resources for plant improvement [45,46,47]. Although doubling of chromosomes does not directly introduce new genetic material, an increase in the number of copies of an allele at a particular site can lead to changes in the process of homologous recombination, which ultimately leads to changes in gene expression and plant phenotypes [48,49]. There are many reports illustrating that polyploidized plants tend to have more biomass and are more resilient than low-ploidy plants. For example, in studies on *Coffea arabica*, although the differences between the homozygous polyploids and the parents were small, genes related to redox activity were more homeostatic in the polyploids, making the polyploidized plants more resistant to stress [50]. Higher resistance to adversity in polyploidized plants was also reported in the study of *Agave americana* by Tamayo et al. [51]. Zhu et al. also reported an increase in stress tolerance in polyploidized plants compared to diploids [52]. Therefore, the study of polyploidy is of great significance for improving plant resistance and germplasm selection. Polyploid plant technology has been widely used in plant breeding, for example, *Arabidopsis thaliana* and *Gerbera jamesonii* [46,53,54]. However, the mechanism of polyploidization on the formation of dominant plant phenotypes needs to be further investigated.

Transcriptomics data analysis can reveal the molecular mechanisms involved in plant growth and development, while metabolomics is the quantitative analysis of all metabolites in an organism. Conjoint analysis of data from the two histologies can improve understanding of biological processes and mechanisms more intuitively and efficiently. In the study of yellowing in chili peppers by Liu et al., the molecular mechanism of leaf green-to-yellow change when exposed to high-intensity light was revealed by the conjoint analysis of transcriptomics and metabolomics data [55]. Conjoint analysis of transcriptomics and non-targeted metabolomics was also used in the study of pepper fruit growth and development and metabolite changes [56]. In this study, we integrated transcriptomics and metabolomics data to analyze DEGs associated with metabolite accumulation and construct a core regulatory network for phenylpropanoid compound synthesis in *C. frutescens* of different ploidy. The metabolic profiles of *C. frutescens* leaves and *C. frutescens* fruits were studied using a widely targeted metabolomics method. A total of six differential metabolites were identified as phenylpropanoids. The accuracy of the RNA-seq data was verified using qRT-PCR [57]. The results showed that the trends of qRT-PCR and RNA-seq were consistent, indicating that the RNA-seq data were of good quality and that RNA-seq data analysis could reflect the differences in gene expression levels between *C. frutescens* at different ploidy levels. The differential gene modes of metabolite regulation identified in this study are consistent with the findings of Liu et al. [58]. In the leaves of *C. frutescens*, the *PAL* gene affects downstream genes through negative regulation, which in turn leads to changes in metabolites. Activation of the *HCT* gene is most pronounced in the fruit of *C. frutescens*, and studies on *Gossypium barbadense* have shown that the *HCT* gene is mainly involved in the biosynthesis of the cell wall [31]. In summary, we speculate that the key genes leading to differences in the production of different ploidy levels of *C. frutescens* may be *PAL* and *HCT*. Balao et al. reported that plant polyploidization will result in changes in quantitative traits such as plant size, stomatal size, etc. [59]. In this study, we observed that the biomass of tetraploid *C. frutescens* leaves, fruits, and whole plants was expanded compared to diploids, and the changes in gene expression levels in the phenylalanine metabolism pathway were more pronounced. We presume that these changes are mainly due to polyploidy.

It has also been reported in previous studies that phytohormone synthesis and signal transduction are the main reasons for the formation of differences in plants of different ploidy. In *Morus alba*, DEGs influence growth hormone and ethylene biosynthesis and signaling, resulting in superior phenotypic traits in tetraploids over diploids [60]. In Shi et al.’s study, chromosome doubling altered the expression levels of genes of the phytohormone signal transduction and the phenylalanine biosynthesis pathway, which ultimately led to changes in the production of metabolite accumulation [61]. In the present study, the doubling of chromosomes led to changes in the accumulation of *C. frutescens* phenylpropanoid compounds, ultimately increasing the biomass of *C. frutescens*. In previous studies of stress tolerance in polyploidized plants, much of the focus has been on analyzing differences in gene expression levels after plant polyploidization [52,62,63]. However, polyploidized plant resistance does not always coincide with changes in gene expression levels, and the mechanisms by which chromosome doubling affects metabolites are more complex [64]. It has been reported in the literature that genome doubling is associated with epigenetic modification variations, such as DNA methylation modifications and protein modifications [65,66,67,68]. Whether *C. frutescens* polyploidy is associated with epigenetic modifications needs further study.

## 4. Materials and Methods

### 4.1. C. frutescens Material, Growth Condition, and Treatment

The *C. frutescens* seeds used in the experiment were conserved by the Germplasm Resources Innovation Laboratory, Southwest Forestry University. Plants were grown in an experimental greenhouse with 15 h of light and 9 h of light avoidance per day. Germinated *C. frutescens* seeds were treated with 1000 mg/L colchicine solution at 4 °C for 24 h and then planted in the experimental greenhouse of Southwest Forestry University. Flow cytometry analysis was performed on the third pair of true leaves 50 d after seed planting using seeds to screen for polyploidized plants, and chromosome filming was performed using the germinated portion of the F1 generation of seeds of the mutagenized material after seed germination. Then, screening was performed again. Morphological comparisons and paraffin sections of the third pair of true leaves 50 d after seed planting were used and stomatal morphology of *C. frutescens* leaves of different ploidy was observed by scanning electron microscopy.

The leaves for polyploid identification were taken at 50 d after seed planting; one leaf was immediately fixed in the fixative solution, and one leaf was immediately frozen in liquid nitrogen and stored at −80 °C for subsequent experiments. Mature fruits were sampled at 80 d after flowering and immediately frozen in liquid nitrogen and stored at −80 °C for subsequent experiments.

### 4.2. Sample Preparation and Extraction

The third pair of true leaves 50 d after seed sowing and mature fruits 80 d after flowering of *C. frutescens* subgeneration that were free of pests and diseases were selected, and the surfaces were rinsed with PBS buffer and immersed in liquid nitrogen for rapid freezing. Then, the materials were stored at −80 °C. The samples were sent to Wuhan Maiwei Metabolic Biotechnology Co., Ltd. (Wuhan, China), for the determination of the composition of phenylpropanoid compounds using the LC-MS/MS technique. Data acquisition was performed by ultra-performance liquid chromatography (UPLC) and tandem mass spectrometry. The liquid chromatography column was ACQUITY BEH C18 1.7 μm, 2.1 mm × 100 mm (Waters Technologies Co., Ltd. Milford, MA, USA). The mobile phases were solvent A (pure water dissolves 0.1% formic acid) and solvent B (acetonitrile dissolves 0.1% formic acid). The elution method was as follows: The starting conditions were 95% solvent A, 5% solvent B. Within 9 min, the gradient was changed linearly to 5% solvent A, 95% solvent B, and the composition of 5% solvent A, 95% solvent B was maintained for 1 min. Subsequently, the components were adjusted to 95% solvent A, 5.0% solvent B within 60 s and held for 180 s. The composition was then adjusted to 95% solvent A, 5.0% solvent B within 60 s and held for 180 s. The liquid phase flow rate was 0.35 mL/min, and the temperature in the column was set at 40 °C.

### 4.3. Differential Metabolites Selected

We used the OPLSR.Anal function in the R package Metabo Analyst R (The software version number is “1.0.1”.) to generate orthogonal partial least squares discriminant analysis (OPLS-DA). The data used were transformed using logarithms (log_2_) and mean-centered before OPLS-DA analysis. To avoid overfitting the data, we also performed a permutation test (200 permutations). In the OPLS-DA analysis of *C. frutescens* leaves and fruits of different ploidy, we identified differential metabolites based on the absolute value of VIP (VIP > 1) values versus Log_2_ FC (|Log_2_FC| ≥ 1.0).

### 4.4. KEGG Annotation and Enrichment Analysis

The differential metabolites identified in the above steps were annotated using the KEGG database (http://www.kegg.jp/kegg/compound/) (accessed on 5 March 2024), and the annotated differential metabolites were mapped to the KEGG database pathway database (http://www.kegg.jp/kegg/pathway.html) (accessed on 6 March 2024) in the KEGG database. The mapped metabolite pathways that were significantly regulated were then entered into MSEA (Metabolite Set Enrichment Analysis), the significance of which was determined by the *p*-value of the hypergeometric test.

### 4.5. Transcriptome Sequencing and Data Analysis

Total RNA was extracted from *C. frutescens* leaf and fruit samples of different ploidy using the CTAB-PBIOZOL method and then identified and quantified using a Qubit fluorescence quantifier (Thermo Fisher Scientific Inc., Waltham, MA, USA) and a Qsep400 high-throughput biofragment analyzer (Vazyme Biotech Co., Ltd., Nanjing, China) to identify and quantify total RNA. Sequencing libraries were constructed using the NEB Next^®^ Ultra™ RNA Library Preparation Kit for Illumina^®^ (New England Biolabs, Inc., Ipswich, MA, USA) by taking 1 μg of RNA from each experimental group versus the control group. After the library construction was completed, a Qubit fluorescence quantifier with Qsep400 high-throughput biofragmentation was used for concentration detection and fragment size detection, and then the effective concentration of the library was accurately quantified using the qRT-PCR method. After the libraries passed the quality control, different libraries were collected according to the effective concentration and the target downstream data volume and then sequenced in PE150 mode (2 × 150 bp) using the Illumina Nova Seq 6000 sequencing platform (Illumina Inc., Santiago, CA, USA). Sequencing data were further analyzed through the Met Ware cloud online platform (http://cloud.metware.cn/) (accessed April–June 2024). Differential expression analysis between sample groups was performed using DESeq2, and a set of differentially expressed genes between the two biological conditions was obtained.

### 4.6. Quantitative Analysis of qRT-PCR

Based on the results of the combined transcriptome and metabolome analysis, genes highly associated with the metabolism of phenylpropanoid compounds (*CCR1*, *CCR2*, *COMT1*, E1.11.1.7 1, E2.1.1.104 1, *PAL1*, *PAL2*, *PAL3*, *4CL1*, and *4CL2*) were screened and their expression patterns were interpreted by qRT-PCR. RNA was extracted from the samples using the Fast- Pure^®^ Plant Total RNA Isolation Kit (Vazyme Biotech Co., Ltd., Nanjing, China) and reverse transcribed into cDNA by the All-In-One 5X RT MasterMix kit (Applied Biological Materials Inc., Shanghai, China) to reverse transcribe RNA into cDNA. The qRT-PCR primers were designed using the primer-blast function in the NCBI database (https://www.ncbi.nlm.nih.gov/) (accessed on 20 June 2024) (Table 2). The UBI gene was used as an internal reference gene for qRT-PCR, and the relative gene expression was calculated using 2^−ΔΔCT^ [23].

### 4.7. Scanning Electron Microscope Analysis

The third pair of true leaves of *C. frutescens* was taken, and the surface stains on the leaves were gently rinsed with PBS (Servicebio Technology Co., Ltd., Wuhan, China) and then quickly placed in the electron microscope fixative ( Servicebio Technology Co., Ltd., Wuhan, China) to be fixed at room temperature for 2 h. The fixed samples were rinsed three times with 0.1 M phosphate buffer PB (PH 7.4) for 15 min each time, and then the tissues were dehydrated in 30%-50%-70%-80%-90%-95%-100%-100% alcohol for 15 min each time and isoamyl acetate for 15 min. Finally, the samples were placed in a critical point dryer for drying and then tightly attached to the conductive carbon film double-sided adhesive, then placed in the sample stage of the ion sputtering instrument for gold spraying for 30 s, and then placed under the scanning electron microscope for observation and image extraction.

### 4.8. Paraffin Section Analysis

The third pair of true leaves of *C. frutescens* was taken and stored in FAA fixative 70% (Servicebio Technology Co., Ltd., Wuhan, China) after gently rinsing the surface stains on the leaves with PBS (Servicebio Technology Co., Ltd., Wuhan, China) quickly. The samples were sent to Servicebio Technology Co., Ltd., Wuhan, China, for paraffin-embedded preparation and analyzed for morphological structural differences.

### 4.9. Chromosome Preparation Analysis

Mature *C. frutescens* seeds were placed on moistened filter paper in a glass Petri dish and placed at room temperature (20–25 °C) for germination. Roots of 1–2 cm in length were collected. Pretreatment was performed using 0.002 mol/L 8-hydroxyquinoline solution for 3 h at 25 °C under dark conditions; after pretreatment, the sample was washed five times with UP (Ultrapure water) water for 2 min each time. The root tips were fixed with Carnotic fixative (anhydrous ethanol: glacial acetic acid: 3:1) for 120 min at 4 °C; after fixation, the root tips were washed with UP water five times for 2 min each time. Root tips were acidolized with 45% glacial acetic acid (*v*/*v*) and a 1 mol/L HCl volume ratio of 1:1 in a water bath at 60 °C for 5 min; after acidolysis was completed, the root tips were washed with UP water for 5 times for 2 min each time, and the root tips were immersed in UP water for 120 min. Staining with carbol fuchsin staining solution for 10–15 min was carried out and then sections were prepared, and then clear chromosome images were searched for under a LEICA DM1000 light microscope (LEICA DM1000, Wetzlar, Germany) at an object lens of 20× and photographed at an object lens of 100×.

### 4.10. Flow Cytometer Analysis

The samples were placed in 0.8 mL of pre-cooled MGb dissociation liquid (45 mM MgCl2-6H2O, 20 mM MOPS, 30 mM sodium citrate, 1% (*w*/*v*) PVP 40, 0.2% (*v*/*v*) Tritonx-100, 10 mM Na2EDTA, 20 μL/mL β-mercaptoethanol, pH 7.5), and with a sharp razor blade the tissues were was rapidly chopped vertically and allowed to stand on ice for 10 min in the dissociation solution and then filtered through a 40 μm pore-size filter to obtain the nucleus suspension. An appropriate volume of pre-cooled propidium iodide (PI) and an appropriate volume of RNAase solution were added to the cell nucleus suspension, and it was placed on ice away from light for 1 h. The working concentration of both the PI staining solution and the RNAase solution was 50 μg/mL [69].

Untreated *C. frutescens* were used as control. Stained cell nuclear suspension samples were up-examined using a BD FACScalibur flow cytometer (BD Biosciences USA, Franklin Lakes, NJ, USA). The fluorescence intensity of the emitted light from the propidium iodide was detected by using a 488 nm blue light excitation, and 10,000 particles were collected for each detection. The coefficient of variation (CV%) was controlled to within 5%. The graphic analysis was performed using Modifit 3.0 analysis software.

## 5. Conclusions

In this study, we investigated the metabolic differences of different ploidy of *C. frutescens* by conjoint analysis of transcriptomics and metabolomics data, focusing on the biosynthetic pathway of phenylpropanoids. A total of six phenylpropanoid differential metabolites were detected to be significantly different by LC-MS analysis. KEGG pathway enrichment analysis revealed significant enrichment of multiple primary and secondary metabolite pathways. Chromosome doubling alters gene expression, ultimately leading to changes in metabolite accumulation. Comprehensive metabolomics and transcriptomics analyses showed that polyploidy down-regulated the expression of phenylpropanoid biosynthesis pathway genes, thereby promoting the biosynthesis of phenylpropanoid compounds. However, specific mechanisms need to be further explored. The results of this study provide a reference for studying the growth differences between different ploidy levels of *C. frutescens*.

## Figures and Tables

**Table 1:** Transcriptome quality control table.

Sampling Place	Sampling Time	Clean Reads	Q30 (%)	Reads Mapped
The third pair of true leaves of diploid	50 d after seeding	7.25–8.04 G	≥95	95.37–95.74%
The third pair of true leaves of tetraploid	50 d after seeding	7.27–7.98 G	≥95	94.85–95.88%
Diploid mature fruit	80 d after flowering	8.38–10.3 G	≥95	96.75–96.91%
Tetraploid mature fruit	80 d after flowering	7.38–10.23 G	≥95	96.71–96.88%

**Table 2:** The primer sequences used in this research.

Gene ID	Gene Name	Forward Primer (5′->3′)	Reverse Primer (5′->3′)
Caz10g11290	*COMT 1*	AAGCCCCACAAATTCCTCGTAT	TAGGTACAACTGGCTCGCAA
Caz02g22970	*E1.11.1.7 1*	GCATCTTTGCTTCTGGATAATAGCA	TTGGTCCACCGGACAGAACA
Caz03g33120	*E2.1.1.104 2*	GAAAGGAGGCTCTGCGGTT	CCGCTGAGCAGAATCCATACA
Caz01g08910	*CCR 2*	CCTTCTTCACCTCAAAGTTTGGAA	AGGTCATTGATCGGTGGCTG
Caz10g10640	*PAL 1*	CTGAGGATGCAAGAGCTGGT	AAACTCCTGCATTCAAGAATCTAAT
Caz09g20200	*PAL 2*	TGCAGCTTTCGAGGACGAAT	AGTTCCAAGTTCCTTCCTCACA
Caz05g22840	*PAL 3*	AGGAAGACAGAGGAGGCACT	GGCTGCTAAACTCACTGCAC
Caz03g05410	*4CL 1*	CACACTGGCGATATGGGGTT	TGCTCGTCTTTCATTGGGAC
Caz03g15840	*4CL 2*	TCAAAGGTTTCCAGGTGCCA	ACTTCCCCTGCAGCATCATC
Caz06g27840	*UBI3*	GTCCATCTGCTCTCTGTTG	CACCCCAAGCACAATAAGAC

## Data Availability

All data are available in the main text. The transcriptome sequence data have been uploaded to the NCBI database, BioProject ID: PRJNA1182981, access link: https://dataview.ncbi.nlm.nih.gov/object/PRJNA1182981?reviewer=r23e3hunlkn7li9k0qf1ocgod4 (accessed on 10 November 2024).
